# Media Use by Children, and Parents’ Views on Children's Media Usage

**DOI:** 10.2196/ijmr.5668

**Published:** 2016-06-07

**Authors:** Meltem Dinleyici, Kursat Bora Carman, Emel Ozturk, Figen Sahin-Dagli

**Affiliations:** ^1^ Eskisehir Osmangazi Univeristy Faculty of Medicine Department of Social Pediatrics Eskisehir Turkey; ^2^ Eskisehir Osmangazi University Faculty of Medicine Department of Pediatric Neurology Eskisehir Turkey; ^3^ Eskisehir Osmangazi University Faculty of Medicine Department of Family Medicine Eskisehir Turkey; ^4^ Gazi University Faculty of Medicine Department of Social Pediatrics Ankara Turkey

**Keywords:** Internet, social media, children, parents, screen time

## Abstract

**Background:**

New (mobile phones, smartphones, tablets, and social media) and traditional media (television) have come to dominate the lives of many children and adolescents. Despite all of this media time and new technology, many parents seem to have few rules regarding the use of media by their children and adolescents.

**Objectives:**

The aim of this study was to evaluate media access/use of children and to evaluate beliefs and attitudes of parents concerning the use of old and new media in Turkey.

**Methods:**

This is a cross-sectional electronic survey of a national convenience sample in Turkey via SurveyMonkey, including 41 questions regarding topics relevant to television, computers, mobile phones, iPad/tablet use, and social media accounts.

**Results:**

The responses of the 333 participants (238 women, 95 men; 27-63 years) were evaluated. The average daily watching alone time was 0 to 2 hours among 53.4% (46/86), and daily coviewing time with parents of children was 0 to 2 hours among 62.7% (54/86) of children below 2 years of age. Regarding parents’ monitoring their children’s computer use (n=178), 35.4% (63/178) of the parents prefer coviewing, 13.5% of the parents use a family filter (24/178), and 33.1% (59/178) of the parents prefer to check Web history. Approximately 71.2% (237/333) of the participants had an iPad/tablet in the house, 84.3% (200/333) of the parents give their children permission to use the iPad/tablet. Of the parents, 22.5% (45/200) noted that their children used the iPad/tablet at the table during lunch/dinner and 57.9% (26/45) of these children were aged 5 years and below. Of parents, 27.3% (91/333) agreed that the optimal age for owning a mobile phone was 12 years, and 18.0% (60/333) of the parents noted that their children (one-third was below 2 years) used the mobile phone at the table during meals. A total of 33.3% (111/333) children/adolescents have a Facebook profile, and 54.0% (60/111) were below 13 years of age. Approximately 89.2% (297/333) of the parents emphasized that the Internet is essential for their child’s education.

**Discussion:**

According to our study results, knowledge regarding the use of old and new media is limited among the parents in Turkey. Our study showed that screen time and mobile device use (including during meals) are common in children below 2 years of age, whereas no screen time was recommended for children below 2 years of age. We concluded that there is need for evidence-based guidelines regarding the use of the Internet and social media for parents and parents should ensure that there is a plan in place for the use of children’s media.

## Introduction

In recent decades, there has been an overload in the diversity of media available. New (mobile phones, smartphones, tablets, and social media) and traditional media (television) have come to dominate the lives of many children and adolescents and the spaces where they spend their leisure time [1,2]. The presence of a computer and/or television in the child’s bedroom and access to the Internet, has also increased; the majority of children and adolescents own mobile phones [1,3,4]. There is an increasing trend among younger children and infants to use mobile devices [5]. The study by Kabali et al [6] showed that children ranging in age from 6 months to 4 years spend 45 minutes a day watching television, 27 minutes watching television shows or videos using a mobile device, 22 minutes using apps on a mobile device, and 15 minutes playing games on a video console. Recently reported “Digital in 2016” of “We Are Social” showed that 58% of whole population in Turkey are active Internet users, 53% are active social media users, 90% have mobile connections, and 45% are active mobile social users [7]. Among the whole population, 86% have a mobile phone (all types), 56% have a smartphone, 48% have a desktop or laptop computer, and 11% have a tablet device. In Turkey, the average daily television viewing time is 2 hours 18 minutes, the average daily use of the Internet via computer or tablet is 4 hours 14 minutes, and the average daily use of social media via any device is 2 hours 32 minutes. Regarding Facebook user profiles, 19% of all users are aged between 13 and 19 years [7].

It is important that parents become aware of the nature of the Internet and social media sites, given that not all of them are healthy environments for children and adolescents. Despite all of this media time and new technology, many parents seem to have few rules regarding the use of media by their children and adolescents [1]. In a recent study, two-thirds of the children and teenagers reported that their parents have “no rules” regarding time spent using social media [3]. There is limited information about the parent’s attitudes in Turkey. The aim of this study was to evaluate media access/use of children, and to evaluate certain beliefs and attitudes of parents about children’s media use including optimal age for use, presence of media in the bedroom, age for having media in the bedroom, time of use, age for opening accounts on social networks, and parental control.

## Methods

### Survey

This study was a cross-sectional electronic survey of a national convenience sample in Turkey. A self-completion questionnaire was developed for parents who use social media to determine their attitudes, beliefs, and opinions concerning the use of mobile phones, television, Internet, and social media of their children. We created a Web-based questionnaire via SurveyMonkey, including 41 questions relevant to topics concerning television, mobile phones, Internet, iPad/tablet use, Facebook, and other social media accounts. A cover letter and questionnaire were electronically mailed via SurveyMonkey to a convenience sample of participants. The questionnaire was mailed with a cover letter explaining the details of the study, identifying the purpose and conﬁdentiality of the study, and reminding potential participants that their participation was voluntary. Anonymity was insured by not requiring names on the questionnaire. No financial incentives were provided for participating in the study

We evaluated the presence of television, computers, mobile phones, and iPad/tablets at home or in the child’s bedroom as well as daily media use of children and adolescents. Our aims also included parents’ views about their children’s media use, and the ways that parents opt to monitor use of media.

### Questionnaire

The questionnaire included questions regarding the following topics: (1) demographic variables (age, gender, educational status, number of children, and age of children), (2) “television” (optimal time for viewing television in the child’s room, presence of television in the participant’s child’s room, age at which the parents allowed the television into the child’s room, average daily watching alone time of children, average daily watching time of children with parents, and preferred and nonpreferred program format for your children), (3) “computer” (optimal time for using the computer in the child’s room, presence of computer in the participant’s child’s room, age at which the parents allowed the computer into the child’s room, presence of Internet connection by the participants in the child’s room, control method of Internet safety/presence of family filter, and use of parent’s computer by the children), (4) “iPad/tablet” (presence of iPad/tablet in the home, parental consent of iPad/tablet use in the home, average daily use of iPad/tablet by the children, and use of iPad/tablet during the child’s meal time), (5) “mobile phone” (optimal time for mobile phone use for children, presence of a mobile phone for the participant’s child, presence of supervision of the child’s mobile phone use, and use of mobile phone during the child’s meal time, (6) “Facebook” (optimal age for opening a Facebook account for children under parental supervision, optimal age for opening a Facebook account for children with their control, presence of a Facebook account for the participant’s child, supervision of the child’s Facebook account by the parents, and attitude concerning the use of child photos by parents in Facebook), and (7) “miscellaneous questions about the use of Internet” (need for use of the Internet for the child’s development and favorite websites and apps for the child’s health and education).

### Data Analysis

A statistical analysis was performed using SurveyMonkey and SPSS 16. The descriptive analysis was calculated within SurveyMonkey and statistical program and described as numbers and percentages.

## Results

### Questionnaire

A self-completion questionnaire was sent via email including a SurveyMonkey questionnaire link to a random selection of 500 people in 2014. In total, 381 participants completed the questionnaire; however, 48 participants were excluded from the study because they had no children.

The responses of the 333 participants (238 women and 95 men) aged between 27 and 63 years (mean age, 38.7 ± 6.1 years) were evaluated. Regarding the educational status of the participants, 44.4% (148/333) were university graduates, and 49.5% (165/333) had PhDs or had achieved a similar level of higher education. A total of 49.8% (166/333) of the participants had one child, 45.0% (150/333) had two children, 4.5% (15/333) had three children, one participant had four children, and one had five children, totally 520 children. The age of the participants’ children varied between 1 month and 18 years; 5.4% (28/520) of the children were below 1 year of age, 11.1 % (58/520) of the children were aged between 1 and 2 years, 16.9% (88/520) of the children were aged between 2 and 5 years, 43.2% (225/520) of the children were aged between 6 and 11 years, and 23.2% (121/520) of the children were aged between 12 and 17 years. Approximately 89.2% (297/333) of the parents emphasized that the Internet is essential for their child’s education.

### Television

According to the participants’ responses, 49.8% (166/333) indicated that there was no need for the presence of television in the child’s room until the child was 18-years old; and 12.2% (41/333) of the participants had no idea regarding the optimal time that television should be available in the child’s room (Table 1). A total of 7.8% (26/333) of participants had a television in their child’s room. Among the all-age group, regardless of the child’s age, the average daily watching alone time of children was 0 to 1 hour according to 42.3% (141/333) of the participants, 1 to 2 hours according to 33.6% (112/333) of the participants, 2 to 4 hours according to 6.3% (21/333) of the participants, >4 hours according to 8.4% (28/333) of the participants, and 9.3% (31/333) of the participants said that they prefer no watching alone. Among the parents who have children below 2 years of age (n=86), the average daily watching alone time of children was 0 to 1 hour according to 33.7% (29/86) of the participants, 1 to 2 hours according to 19.7% (17/86) of the participants, 2 to 4 hours according to 15.1% (13/86) of the participants, >4 hours according to 4.6% (4/86) of the participants, and 26.7% (23/86) of the participants said that they prefer no watching alone. Among the parents who have children aged between 2 and 17 years of age (n=267), the average daily watching alone time of children was 0 to 1 hour according to 42.6% (114/267) of the participants, 1 to 2 hours according to 35.2% (94/267) of the participants, 2 to 4 hours according to 5.2% (14/267), >4 hours according to 9.8% (26/267) of the participants, and 7.2% (19/267) of the participants said that they prefer no watching alone.

The average daily watching time of children with parents (coviewing) was 0 to 1 hour according to 28.2% (94/333) of the participants, 1 to 2 hours according to 36.0% (120/333) of the participants, 2 to 4 hours according to 18.6% (62/333) of the participants, >4 hours according to 12.0% (40/333) of the participants, and 4.8% (16/333) of the participants said that they prefer no watching alone. Among the children below 2 years of age, the average daily coviewing time was 0 to 1 hour according to 38.3% (33/86) of the participants, 1 to 2 hours according to 24.4% (21/86) of the participants, 2 to 4 hours according to 8.1% (7/86) of the participants, >4 hours according to 10.4% (9/86) of the participants, and 18.6% (16/86) of the participants said that they prefer no watching alone. Among the children aged between 2 and 17 years, the average daily coviewing time was 0 to 1 hour according to 26.5% (71/267) of the participants, 1 to 2 hours according to 37.8% (101/267) of the participants, 2 to 4 hours according to 20.2% (54/267) of the participants, >4 hours according to 11.9% (32/267) of the participants, and 3.3% (9/267) of the participants said that they prefer no watching alone.

**Table 1:** Belief and Attitudes of Parents About Television Use During Childhood

Television		n	%	95% confidence interval
**Optimal time to presence of television in child’s room**				
	No television in child’s room until 18	166	49.8%	44.3-55.1
	<2 years	0	-	-
	2-5 years	8	2.4%	0.8-4.0
	6-11 years	37	11.3%	7.9-14.7
	12-17 years	81	24.3%	19.7-28.9
	Not known	41	12.2%	8.7-15.7
**Presence of television in the child’s bedroom**				
	Total	26	7.8%	4.9, 10.7
	0-2 years	1	3.8%	0, 11.5
	2-5 years	2	7.6%	0, 17.8
	6-11 years	11	42.3%	23.3, 61.3
	12-17 years	12	46.1%	26.9, 65.2

### Computer

According to the participants’ responses, the optimal time for the presence of a computer in the child’s room varies between 1 and 18 years. Table 2 summarized the results of parental beliefs and children use of computer. The optimal time has been defined as 6 to 10 years by 38.9% (123/333) of parents and 11 to 14 years by 24.0% (80/333) parents, 17.4% (58/333) of the participants had no idea, and 6.9% (23/333) of the participants thought that there was no need for a computer to be available in the room of a child below 18 years of age. Approximately 28.0% (82/333) of the participants have a computer in the child’s room, and the age at which the parents brought the computer into the child’s room varies from 1 to 17 years (median age, 9 years). Approximately 95.1% (78/82) of the children who have a computer in their room also have an Internet connection via a wireless modem in the house. Regarding the parents’ monitoring of their child’s computer use, 35.4% (63/178) of the parents prefer coviewing, 13.5% (24/178) of the parents use a family filter, 33.1% (59/178) of the parents prefer to check Web history, and 17.9% (32/178) of the participants do not monitor their child’s computer use (Table 2).

**Table 2:** Belief and Attitudes of Parents About Computer Use During Childhood

Computer		n	%	95% confidence interval
**Optimal time to presence of computer in child’s room**				
	No computer in child’s room until 18	23	6.9%	4.2, 9.6
	1-5 years	16	6.3%	3.7, 8.9
	6-10 years	123	36.9%	31.7, 42.1
	11-14 years	80	24.0%	19.4, 28.6
	15-17 years	33	9.9%	6.7, 13.1
	Not known	58	17.4%	13.3, 21.4
**Presence of computer in the child’s bedroom**		82	27.8%	23, 32.6
	1-2 years	2	2.4%	0, 5.7
	2-5 years	2	2.4%	0, 5.7
	6-11 years	34	41.5%	30.8, 52.1
	12-17 years	44	53.7%	42.9, 64.5
**Presence of monitoring method for child’s computer use**		178		
	Coviewing	63	35.4%	28.3, 42.4
	Family filter	24	13.5%	8.4, 18.5
	No monitoring	32	17.9%	12.2, 23.5
	Check website history	59	33.1%	26.1, 40.0

### iPad/Tablet

Approximately 71.2% (237/333) of the participants have an iPad/tablet in the house, and 84.3% (200/237) give their children permission to use the iPad/tablet. The age distribution of the children were summarized in Table 3. Among the all-age group, the average daily iPad/tablet use of children was 0 to 1 hour according to 63.9% (213/333) of the participants and 1 to 2 hours according to 16.8% (56/333) of the participants. Among the children below 2 years of age, the average daily iPad/tablet use of children was 0 to 1 hour according to 59.3% (9/15) of the participants. Among the children aged between 2 and 17 years of age, the average daily iPad/tablet use of children was 0 to 1 hour according to 64.7% (120/185) of the participants, 1 to 2 hours according to 18.9% (35/185) of the participants, 2 to 4 hours according to 5.4% (10/185) of the participants, and more than 4 hours according to 10.8% (20/185) of the participants.

Approximately 22.5% (45/200) of the parents noted that their children used the iPad/tablet at the table during lunch/dinner. Of these children, 26.6% (12/45) were aged between 0 and 2 years, 31.1% (14/45) of the children were aged between 2 and 5 years (Table 3).

**Table 3:** Belief and Attitudes of Parents About iPad/Tablets Use During Childhood

iPad/Tablet		n	%	95% confidence interval
**Presence of iPad/tablet at home**		237	71.2%	66.3, 76.0
**Child’s use of iPad/tablet at home**		200	84.3%	79.7, 88.9
	0-1 years	2	1.0%	0, 2.4
	1-2 years	13	6.5%	3.0, 9.9
	2-5 years	31	15.5%	10.5, 20.5
	6-11 years	111	55.5%	48.6, 62.3
	12-17 years	43	21.5%	15.8, 27.2
**Use of iPad/tablet during lunch/dinner**		45	22.5%	16.7, 28.2
	0-1 years	1	2.4%	0, 6.8
	1-2 years	11	24.4%	11.9, 37
	2-5 years	14	31.1%	17.8, 44.6
	6-11 years	12	26.7%	13.7, 39.6
	12-17 years	7	15.6%	5.0, 26.2

### Mobile Phone

According to the participants’ responses, 3.3% (11/333) indicated that there was no need for the presence of a mobile phone for children until the age of 18 years. Approximately 19.5% (65/333) of the participants responded as 6 to 11 years, 59.8% (199/333) of the participants responded as 12 to 17 years (Table 4). According to the participants’ responses, 27.3% (91/333) indicated that the optimal time for owning a mobile phone was 12 years of age. Parents commonly bought their children a mobile phone when they were more than 7-years old, and 17.4% (58/333) of the participants thought that the optimal time for owning a mobile phone was when their children started to attend college. A total of 91 children had mobile phones during the study period, and among them, 64.8% (59/91) are between the ages of 12 and 17 years. Approximately 18.0% (60/333) of the parents noted that their children used a mobile phone at meal times or crying and 6.7% (4/60) were less than 1-year old, 23.3% (14/60) of these children ranged in age between 1 and 2 years, 26.7% (16/60) were aged between 2 and 5 years, 36.7% (22/60) were aged between 6 and 11 years, and 6.8% (4/6) were more than 11-years old (Table 4).

**Table 4:** Belief and Attitudes of Parents About Mobile Phone Use During Childhood

Mobile phone		N	%	95% confidence interval
**Optimal time to presence of mobile phone (children)**				
	No mobile phone until 18	11	3.3%	1.4, 5.2
	<2 years	-	-	-
	2-5 years	-	-	-
	6-11 years	65	19.5%	15.2, 23.8
	12-17 years	199	59.8%	54.5, 65.1
	Not known	58	17.4%	13.3, 21.4
**Percentage of children who have a mobile phone**		91	27.3%	22.5-32.1
	1-2 years	2	2.2%	0, 5.2
	2-5 years	1	1.1%	0, 3.2
	6-11 years	29	31.9%	22.3, 41.4
	12-17 years	59	64.8%	54.9, 74.6
**Use of mobile phone during lunch/dinner or crying**		60	18.0%	13.8, 22.1
	0-1 years	4	6.7%;	0.37, 13.0
	1-2 years	14	23.3%,	12.6, 34.0
	2-5 years	16	26.7%;	15.5, 37.9
	6-11 years	22	36.7%;	24.5, 48.9
	12-17 years	4	6.7%;	0.37, 13.0

### Facebook

According to the participants’ responses, the median optimal age for opening a Facebook account for children under parental supervision was 8 years; the optimal age for a Facebook account for children with their control was 13 years. Regarding the participants’ response, the optimal age for a Facebook account for their children under parental supervision was below 5 years (6/333, 1.8%), 5 to 12 years (114/333, 34.2%), 13 to 17 years (117/333, 34.8), and above 18 years (22/333, 6.6 %).

Table 5 summarized the optimal age for opening a Facebook account for their children with their control. Of 85 participants 25.5% (22/85) had no idea regarding the optimal age for opening a Facebook account for their children under their control. A total of 111 children/adolescents have Facebook profiles; 53.2% (60/111) of them are below 13 years of age. When we evaluated the monitoring method for childhood Facebook, 16.2% (18/111) of the parents prefer “coviewing,” 45.0% (50/111) of the parents prefer their children’s Facebook account share the same password, and 32.4% (36/111) of the parents have no monitoring method.

**Table 5:** Belief and Attitudes of Parents About Facebook Use During Childhood

Facebook		N	%	95% confidence interval
**Optimal time for presence of Facebook account for children under the parent’s supervision**				
	0-5 years	6	1.8%	0.37, 3.2
	5-12 years	114	34.2%	29.1, 39.3
	13-17 years	117	34.8%	29.6, 39.9
	18 years/above	22	6.6%	3.9, 9.2
	Not known	75	22.5%	18.0-26.9
**Optimal time for presence of Facebook account for children with their control**				
	0-5 years	1	0.3%	0, 2.0
	5-12 years	29	8.7%	5.7, 11.7
	13-17 years	124	37.2%	32.0, 42.4
	18 years/above	94	28.3%	23.4, 33.11
	Not known	85	25.5%	20.8, 30.1
**Presence of child’s Facebook account**		111	33.3%	28.2, 38.3
	0-1 years	3	2.7%	0, 5.7
	1-2 years	3	1.8%	0, 4.2
	2-5 years	8	7.2%	2.4, 12.0
	6-11 years	46	41.4%	32.2, 50.5
	12-17 years	52	46.8%	37.5, 56.1
**Monitoring method for child’s Facebook account**		111		
	Coviewing	18	16.2%	9.3, 23.1
	Facebook friend with child	7	6.3%	1.8, 10.8
	No monitoring	36	32.4%	23.7, 41.1
	Check child’s account	50	45.0%	35.8, 54.2

## Discussion

### Strengths of This Study

To the best of our knowledge, this study is the first to evaluate knowledge regarding the use of media relative to parents and their children in Turkey. This study showed that there is widespread access and use of media by children of all ages and the majority of the participating parents have limited information regarding this subject.

### Television

According to the participants’ responses, 50.0% (166/333) of them thought that the television should be kept out of the child’s bedroom if the child is below 18 years of age. Approximately 8.0% (26/333) of the participants’ children have a television in their bedroom, and 50.0% (13/26) of them are below 10 years of age. The average daily watching alone time was less than 2 hours among 75.9% (253/333) of the participants; however, 55.0% of the children under the age of 2 years watch television <2 hours. The American Academy of Pediatrics (AAP) recommends limiting the amount of total entertainment screen time to <1 to 2 hours per day (above 2 years and no screen below 2 years of age), keeping the television out of the child’s bedroom, and to coview television with children and adolescents [1]. Parents should watch television with their children to teach them how to interpret the media messages or the content of commercials. Parental supervision during watching enables the children to distinguish between reality and fantasy [7]. Several studies have documented that higher levels of screen time are associated with less sleep, more attention problems, and lower academic performance [8-10]. Tomopoulos et al [11] showed that young children under age two frequently watch background media that has age-inappropriate content or has not been turned on for them to watch. Brockmann et al [12] showed that the presence of a television in the child’s bedroom was associated with significantly reduced sleep quality, “sleep terrors,” “nightmares,” and “sleep talking” among children aged between 1 to 4 years. Two-year-old children in the United States watch an average of 2 hours of television each day, with nearly half watching more than that amount. Australian 2- to 4-year-old children participate in 83 minute per day of electronic media use, and only 26% of those children meet the Australian recommendation [5,13]. In our study, approximately 65% of the participants meet the AAP recommendations for watching television, which is higher than in other countries and this result might be related our study population, which includes parents with high-education levels.

### Computer

In our study, the parents have a lack of knowledge regarding the optimal time for computer presence in the child’s bedroom, and only 6.9% (23/333) of the participants thought that there was no need for a computer to be present in their child’s room. Approximately 28% (82/333) of these study participants have a computer in the child’s room, and the age at which the parents brought the computer into the child’s room was a median of 9 years; the majority of the children have WiFi access in their bedroom. Approximately 82.0% (146/178) of the parents employ safety monitoring for supervision including coviewing, using a family filter or checking their child’s website history. In the United States, nearly one-third of TV programming is viewed on alternative platforms in computers, iPads, or mobile phones, nearly all have Internet access, and one-third have access to a variety of social media in their own bedroom [1]. Children and adolescents spend up to 1.5 hours per day with their computer; half of this time is spent social networking, playing games, or viewing videos. The AAP has recommended keeping Internet-connected electronic devices out of the child’s bedroom and monitoring what type of media their children are using [1].

### iPad/Tablets

In our study, 84.3% (200/237) of the parents who have iPads/tablets give their children permission to use the iPads/tablets; 81.0% of the children used the devices less than 2 hours per day, whereas 59.3% of the children aged below 2 years used iPad/tablets approximately 1 hour. Nearly 22.5% (45/237) of the parents noted that their children used iPads/tablets at the table during meal time; 26.6% (12/45) of these children were aged below 2 years. Recent recommendations discourage screen media exposure for children <2 years of age and also enforcing a meal time “curfew” for media devices [1]. Contrary to the recommendations regarding television, the parents have no knowledge about the use of new media devices, such as tablets. The AAP recommendations for media use have been maintained before the first generation iPad and the overload of apps aimed at young children [6,14]. In the United States, more than 30% of young children play with a mobile device, as mentioned in our study [6,15]. In the United States, a recent survey among children aged 6 months to 4 years showed that 96.6% of the children had used a mobile device; the devices were mainly iPads and the majority of the parents let their children play with the mobile devices while they did chores (70%), to keep the child calm in public places (65%), while running errands (58%), or to put their child to sleep (28%) [6]. Parents gave or took away mobile devices to reward or punish their child’s behavior, using it as a “digital pacifier” to placate or distract children or as a means to manage their children’s behavior [6]. For this reason, new recommendations are required for use of iPads/tablets and apps for children.

### Mobile Phone

There was no consensus among parents regarding the optimum time for mobile phone use; they commonly bought mobile phones for their children older than 7 years of age. In our study, 18.0% (60/333) of the parents noted that their children used mobile phones at the table during meal times or crying and that 30.0% (15/60) of the children were below 2 years of age. AAP recommended that to build a model for active parenting, it was essential to establish a family home use plan for all types of media, including cell phones, and establish reasonable but ﬁrm rules relative to cell phones and texting [1]. The younger children also like to use multiple media platforms, such as television and tablets, at the same time [6]. Approximately 50% of the parents of children aged 6 months to 4 years downloaded apps (educational, entertainment, and content delivery) on their mobile devices mainly for their children. Excessive media use is not only a problem in Western countries; the results of the national school violence study in South Africa showed that 80% of secondary school learners have a mobile phone. Approximately 70% of these children were reported to use social network sites and talk with strangers at least once a week [15].

### Social Media

According to the participants’ responses, the majority of participants have no idea about the optimal age for opening a Facebook account. Barbovschi et al [16] showed that 42% of children aged 9 to 12 years have profiles on Facebook, many with the explicit permission of their parent, despite the explicit policy between 2012 and 2014 from the Net Children Go Mobile (NCGM) project allowing only children aged 13 years and older to have a profile on Facebook. Among the children ranging in age between 9 and 12 years, Facebook use was connected to daily use of the Internet from home, looking for new friends online [16]. In our study, 53.0% (60/111) of the children who have Facebook accounts were below 13 years of age, which was similar to the NCGM project. In our study, parents brought the computer into the child’s room at a median age of 9 years, and the majority of the children have WiFi access in their bedroom. Although Facebook has an explicit policy that allows only children older than 13 years of age to have a profile, parents should supervise Internet activity, including the social media accounts of their children. According to the Eurokids Online study results Turkey is categorized as a “low use, some risk” country [17]. Eurokids Online report emphasized that because many Turkish children are heavily dependent on out-of-home Internet access, the parents may find it difficult to regulate their children’s Internet use. Approximately 15% of the children in Turkey have visited at least one harmful website (websites related to committing suicide, being anorexic, or hate groups, etc), whereas the incidence in Europe is 18% [17].

### Media Use and Health Effect

The current international recommendations are that children aged 2 to 5 years should engage in 1 hour or less of electronic media use (television/digital versatile disc/video, computer use, and electronic games) per day [1,5]. In our study, we observed that screen time and mobile device use are common in children below 2 years of age. Higher levels of early childhood electronic media use are associated with children being at risk for poor outcomes in several indicators of well-being. Hinkley and colleagues [5] emphasized that participation in high levels of electronic media use during early childhood (more than 2 hours per day) has been linked with increased weight status, behavioral problems, poor language and cognitive development, and poor social competence. Increased weight and obesity might be associated with television viewing and sedentary behaviors during childhood, and both of these conditions coexist with multiple other unhealthy behaviors, including poor dietary behaviors [5,18]. Falbe et al [19] showed that each hour-per-day screen time increase was associated with increased intake of total foods of low nutritional quality, increased intakes of sugar-sweetened beverages, fast food, sweets, and salty snacks, and decreased intakes of fruits and vegetables. It is important to encourage families to monitor their children’s media use and spend more time doing physical activities with their children to improve cardiovascular health in their adulthood [7]. There are contradictory results regarding the effect of electronic media use, and there is a two-sided coin that may affect both physical and psychological health [2]. Boniel-Nissim et al [20] evaluated the international trends in electronic media communication among the 11- to 15-year olds in 30 countries from 2002 to 2010. They mentioned that although Internet usage is often blamed for its negative effects on teenagers’ social interactions in the physical world, an electronic device was found to predict ease of communication with friends as a powerful tool for helping people to connect.

### Limitations

We studied parents with social media access, we realized that this population may not represent the entire population, and we determined that our findings may not be generalizable. Some results about the use of mobile device are not credible and are probably biased by the Web-based questionnaire administration. A higher percentage use of technology might be more likely to complete the Web-based survey and are the most likely to have mobile devices at home. Our study does not evaluate the impact that mobile media devices have on children; we have provided a recent situation concerning the usage of media devices among children. This study also demonstrated the need for a guide that includes recommendations for both health care providers and families on the use of mobile media by young children.

### Conclusion

According to our study results, there is limited knowledge regarding the use of old and new media among parents in Turkey. Mobile phones, tablet computers, and social media are widely used by children, especially in the age group where electronic media use should be discouraged. However, there is a lack of sufficient research and guidelines on protecting children’s safety in the use of media devices in developing countries, as well as in Turkey. Therefore, physicians, especially pediatricians, should make parents and teachers media-literate, meaning that pediatricians should comprehend the risks of media exposure because they are uniquely positioned to provide scientifically based recommendations to families [7,21]. Encouraging parents to monitor children’s media carefully can have a wide range of health benefits for children. Specifically, there are four types of parental monitoring: (1) coviewing with the child, (2) restricting amount of media use time, (3) restricting the types of content, and (4) actively discussing the meanings and effects of media content with children (active mediation). Several studies have found that coviewing paired with active mediation, restricting amount of media exposure, and restricting content are all powerful protective factors for children.

 Active mediation can include offering opinions of media content, educating children regarding the purposes of various media (eg, advertising), or providing guidance and explanations [21]. Gentile et al [22] evaluated the prospective effects of parental monitoring of children’s media on the physical, social, and academic outcomes. They showed that parental monitoring of children’s media influences children’s sleep, school performance, and prosocial and aggressive behaviors, and that these effects are mediated through total screen time and exposure to media violence. Parental monitoring of media has protective effects on a wide variety of academic, social, and physical child outcomes. According to the results obtained from our study, we concluded that there is a need for evidence-based guidelines on the use of the Internet and social media for parents. Parents should ensure that there is a plan for the use of children’s media.

## Abbreviations

AAP: American Academy of Pediatrics
NCGM: Net Children Go Mobile
